# FAME, the Flux Analysis and Modeling Environment

**DOI:** 10.1186/1752-0509-6-8

**Published:** 2012-01-30

**Authors:** Joost Boele, Brett G Olivier, Bas Teusink

**Affiliations:** 1 Systems Bioinformatics/AIMMS, VU University Amsterdam, De Boelelaan 1085, 1081HV, Amsterdam, The Netherlands; 2 Netherlands Institute for Systems Biology (NISB), Amsterdam, The Netherlands; 3 Life Sciences, Centrum voor Wiskunde en Informatica (CWI), Science Park 123, 1098XG, Amsterdam, The Netherlands

## Abstract

**Background:**

The creation and modification of genome-scale metabolic models is a task that requires specialized software tools. While these are available, subsequently running or visualizing a model often relies on disjoint code, which adds additional actions to the analysis routine and, in our experience, renders these applications suboptimal for routine use by (systems) biologists.

**Results:**

The Flux Analysis and Modeling Environment (FAME) is the first web-based modeling tool that combines the tasks of creating, editing, running, and analyzing/visualizing stoichiometric models into a single program. Analysis results can be automatically superimposed on familiar KEGG-like maps. FAME is written in PHP and uses the Python-based PySCeS-CBM for its linear solving capabilities. It comes with a comprehensive manual and a quick-start tutorial, and can be accessed online at http://f-a-m-e.org/.

**Conclusions:**

With FAME, we present the community with an open source, user-friendly, web-based "one stop shop" for stoichiometric modeling. We expect the application will be of substantial use to investigators and educators alike.

## Background

In the post-genome era, genome-scale stoichiometric models have gained popularity, as the absence of the need to experimentally determine model parameters one enzyme at a time made it possible to build bigger metabolic models than ever before [1]. However, genome-scale stoichiometric models quickly get so large that efficiently editing them requires advanced tools. It is not even a trivial matter to run a model and visualize the results, as this requires at least a linear solver and some program to interpret the solver's output.

Although tools that facilitate common tasks are available (e.g. Model SEED for automated model generation [2], the COBRA Toolbox for model solving and command-line manipulation [3]), and some cater to more than one need (e.g. OptFlux [4] or the Java-based CellNetAnalyzer [5], which both feature model editing and a form of visualization), none have proven to be the panacea that bridges these gaps. Broad adoption of tools is often impeded by complicated installation procedures, required proprietary software (e.g. Matlab), not scaling up to genome-scale proportions, or results visualization that requires extensive user input in order to produce intelligible results.

We identified and experienced the need for an open-source, user-friendly and portable (web-based) software environment for most routine questions a (systems) biologist would want to ask a genome-scale metabolic model. Based on our own extensive experience in developing and using such models, we have developed FAME: the Flux Analysis and Modeling Environment, a "one stop shop" that addresses these issues.

### Comparison with existing tools

In an analysis of available applications, the programs that approach FAME's functionality the closest are the aforementioned COBRA Toolbox, OptFlux, and CellNetAnalyzer. We will discuss these tools here, but also refer to Table 1, where we have summarized a more complete assessment of the alternatives.

**Table 1:** Comparison between FAME and existing software

Tool name	**Ref**.	Visualization			Run				Oper. mode		Model handling			
					
		Supervised	Unsupervised	User-supplied	FBA	FVA	MOMA	Gene/rxn	Stand-alone	Web-based	Build	Edit	Import SBML	Export SBML
FAME		●		●	●	●		●		●	●	●	●	●
Model SEED	[2]	●			●			○^1^		●	●	○^2^		●
COBRA Toolbox	[3]			●	●	●	●	●			●	●	○^3^	○^3^
OptFlux	[4]			●	●	●	●	●	●				●	●
CellNetAnalyzer	[5]			●	●	●							●	●
PySCeS	[7]				●	●			○^4^			●	●	●
YANASquare	[13]		●	●	●				●		●	●	●	
MEGU	[14]	●								●				
BioMet Toolbox	[15]				●			●	○^4^	●				
Cytoscape	[16]		●	●				○^1^	●		●	●		

Notes:

1. Gene/reaction associations are used to build models, but no analyses are performed on them.

2. Model SEED has only limited editing capabilities once a model has been produced.

3. The COBRA Toolbox uses an SBML dialect specific to this tool.

4. Certain dependencies must be installed first.

A comparison of the features offered in FAME and existing alternatives. A ● indicates a feature that is present; an open circle (○) denotes a partial implementation of the feature in question. Under visualization, "supervised" means the application uses predefined network topologies, "unsupervised" means networks are drawn on the fly, as graphs, and "user-supplied" means that visualization is performed on user-supplied network topology maps.

Under "Run", MOMA represents Minimization Of Metabolic Adjustments, which is described in [17]. Most packages feature additional analyses besides the ones listed in this table. As many of these tools are custom-built for these analyses, or vice versa, we do not list those analysis options in this table. The tools whose functionalities most resemble FAME's have no web interface or have inferior visualization capabilities. On the other hand, tools featuring (or focusing on) superior visualization lack the powerful analysis features unlocked by PySCeS-CBM.

The COBRA Toolbox [3] is one of the most widely used toolkits for (stoichiometric) systems biology modeling. It has a very complete editing and analysis feature set, and features results visualization on user-supplied network maps. Although the toolbox itself is open-source, it is dependent on Matlab, which may deter impecunious users. Moreover, to perform any routine tasks or data analysis, users must first learn to use Matlab.

OptFlux [4] and CellNetAnalyzer [5] are tools that integrate some or all of FAME's key functionalities, particularly model editing and visualization. However, neither tool has a web interface, and CellNetAnalyzer is based on Matlab, which makes it suffer from similar limitations as the COBRA Toolbox. In both tools, as well as in the COBRA Toolbox, visualization is dependent on user input of the network topology in a tool-specific format, such as a CellDesigner [6] map or COBRA Toolbox-specific "map file". FAME offers supervised visualization in a web interface, and this can be considered an enhancement of existing functionality in three ways: first, users need not supply a custom-made map file to visualize results; second, FAME scours models for meta-information that might aid in the visualization of run results (e.g. EC-numbers); and third, FAME uses this information to generate maps that are interactive, with elements that can be clicked to access additional information. It is the first application to open up this feature set in an installation-free manner, and to harness the functionality of the web for this kind of analysis.

## Implementation

Besides the externally visible HTML and CSS that convey its markup, the parts of the FAME web application that do the work are implemented in PHP5 and JavaScript. PHP was chosen because it is a fast, browser independent language that integrates well with other programs. FAME uses the Python-based PySCeS-CBM (Python Simulator of Cellular Systems-Constraint Based Modelling toolkit) [7] as an interface to a linear solver. Model information, which is encoded in XML, is communicated to PySCeS using SOAP, the Simple Object Access Protocol. Run results are retrieved over the same protocol, and this setup of FAME as a SOAP client opens the door for it to gather data from a variety of resources in future releases (e.g. the Kyoto Encyclopedia of Genes and Genomes (KEGG, by Kanehisa and Goto [8])). Moreover, as both FAME and PySCeS have a modular architecture, future expansions of the analysis capabilities of PySCeS-CBM will conveniently translate to cognate expansions of FAME's functionality. When visualizing, FAME generates pathway maps as SVG images (Scalable Vector Graphics), using an algorithm designed specifically for FAME. The SVG format was selected because of its open nature (it is an XML-based format), cross-platform compatibility and scalability. In addition, SVG images natively support the inclusion of hyperlinks, which adds a layer of interactivity to run results. FAME, PySCeS(-CBM), and Mariner, the SOAP interface to PySCeS, are all open source.

## Results and Discussion

**Figure 1:**
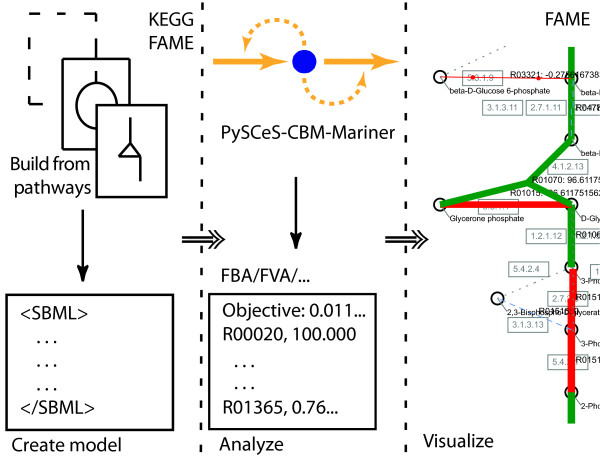
**General FAME work flow**. FAME's three key functionalities: model creation, result generation, and visualization/interpretation. The dashed lines between panels mark the points where users would switch to different programs to perform different tasks. By eliminating these switching points, work flow is streamlined.

**Figure 2:**
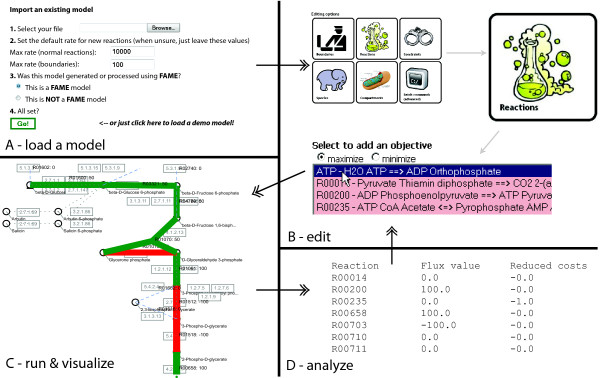
**Example work flow**. A demonstration of practical usage of FAME. This example illustrates the addition of an objective function to a model of glycolysis, and the model's subsequent execution. (a) On the "Import existing model" screen, users can load SBML models with or without constraints. In the latter case, constraints information will be added by FAME and can be changed later. By just clicking "Go!" without supplying a model, the demo model described in this paper will be loaded. (b) Six buttons provide access to the various model editing options. Advanced users are able to enter batch commands to edit (and run) the model ("Batch commands" button), but it is also possible to edit in a graphical interface (the other five buttons). In this example, an objective is added by clicking "Reactions", selecting the desired objective reaction, and applying the changes. (c) When the model is ready to run, clicking the "Run!" button will produce the results in both tabular and graphical formats. (d) Results can be exported as tab separated values files for further analysis, or, alternatively, more detailed metabolite-centered results representations can be viewed.

A web-based application, FAME aims to address the needs of modelers on three key points of focus: *model creation*, *result generation*, and *interpretation *(i.e. visualization, sensitivity analysis, metabolite connectivity, etc.) (Figure 1). Traditionally, transitions between these tasks often impeded work flow and could themselves become a source of errors. For example, running a model after editing it, or visualizing the results after running it, would require the user to save a file, launch another program, and then load the file into the new program. In FAME, these labor-intensive transitions are eliminated by teaming up with Mariner, a SOAP interface of PySCeS-CBM [7]. Throughout this section, we will illustrate FAME's functionality based on an example use case (Figure 2).

### Creating and editing models

FAME allows users to either upload their own preexisting model (Figure 2A), or to build a new model based on the information in KEGG. When building from scratch, it is possible to select a subset of pathways from KEGG, foregoing the inclusion of unnecessary reactions that may be present in existing genome-scale reconstructions. To allow for fast model construction, FAME uses a cached copy of the required information from KEGG for the assembly of new models. In addition, the KEGG IDs in such models can be used to find more information from KEGG if the need should arise. FAME's visualization module makes use of these IDs when mapping run results onto KEGG maps. Importing non-KEGG, non-FAME models may inactivate some of these capabilities, although measures have been taken to use metadata that is available in imported models, for example the models from the BiGG database [9]. As an alternative to building from scratch, any stoichiometric model can be loaded into FAME, provided it is encoded in the Systems Biology Mark-up Language (SBML, [10]), the *de facto *standard for representing such models. A proposed SBML Level 3 package "Flux Balance Constraints" allows the definition of constraint based models [11]. Models that lack information about constraints, however, will also load in FAME, and will be automatically converted to constraint based models as necessary. FAME is intentionally very flexible with respect to the integrity of the input SBML, accepting even a bare minimum of information about a model's stoichiometry.

Once a model is loaded, FAME offers all tools a seasoned constraint-based modeler would need to study physiology. This includes easy editing of the flux bounds on all internal and exchange reactions, editing existing reactions, adding/deleting objectives, recognizing dead-end metabolites (orphans), recognizing synonymous reactions, and assigning reactions to different or new compartments (Figure 2B). Adding exchange reactions is supported, as are performing operations in batch and setting constraints on a per-reaction basis. The current version of a model can be exported as SBML at any time.

### Result generation

Analysis commands are forwarded to PySCeS-CBM, which handles the mathematical operations and returns the results to FAME. Operations supported by PySCeS include Flux Balance Analysis (FBA) and Flux Variability Analysis (FVA). Given the more compute-intensive nature of the latter, if a subset of pathways is selected, only reactions in those pathways will be included in the variability analysis. For instance, whereas an FBA of the *S. cerevisiae *model [12] from BiGG (1266 reactions) takes under ten seconds (including visualization), the equivalent FVA takes roughly ten times as long. In addition, FAME can minimize the sum of absolute fluxes of an FBA solution, which leads to results that are more biologically relevant, as it can reduce complex loops in FBA solutions to their underlying net fluxes. FAME can also perform analyses on metabolites, rather than reactions or fluxes: per metabolite, it can list right hand side sensitivities, shadow prices, and it also features the option of checking whether a specific metabolite can be produced by the model. The latter can also be performed for all metabolites in the model at once. Once generated, results can be visualized, but they are also always presented as a human-readable table (which includes reduced costs for each reaction) and as a machine-readable, tab-separated format file that can be imported in e.g. Excel (Figure 2D).

The included Gene Association Workbench allows users to intuitively take advantage of gene association information present in the model metadata, e.g. by simulating (multiple) knockout mutants. Results are presented in the same interactive manner as the other analyses. If on any occasion the model system is over-determined, FAME will relay the solver's message that the solution status is 'infeasible' and additionally issues a warning to the user. Under-determined systems will run and produce a result; upon interpreting run results, users may run further analyses such as FVA to assess the properties of the solution space.

### Visualization

The visualization module generates images in SVG format, based on the analysis results returned by PySCeS. The advantages of using SVG are manifold, some of the more notable being image scalability and ease of editing using third party software. Depending on the web browser used, users may need to download a (free) plug-in to view the images.

For each selected pathway an interactive KEGG-like image is drawn (Figure 2C), on which the run results are superimposed. To the biologist, this readily recognizable representation is an improvement over unsupervised visualization algorithms (e.g. in [13]), and while this approach to data visualization was already applied some years ago [14], to our knowledge, FAME is the first web-based application that both generates data and automatically visualizes results.

Many elements in the results images are clickable (another advantage of the SVG format), to make more information available more conveniently. For instance, clicking a metabolite will display an overview of reactions producing or consuming it, along with the KEGG information page for the metabolite, while clicking a reaction will display that reaction's KEGG information page.

## Conclusions

With FAME, we present the community with an easy to use web-based "one stop shop" for the manipulation and execution of stoichiometric models. It enables biologists to create or import models, edit them, run them at the click of a button, and visualize the results from the browser window. We expect that its install-free integration of execution and visualization will appeal to investigators and educators alike. Future releases of FAME will feature integration with web-based annotation services and further analysis options. Finally, the novel SOAP interface to PySCeS-CBM will facilitate the creation of user-friendly interfaces based on PySCeS that will uncover powerful modeling functions that may otherwise remain hidden behind the ever-enigmatic command line cursor.

## Availability and Requirements

FAME is intended and offered as a web service, but can also be installed locally, as source code will be made available upon request. FAME can be accessed online at http://f-a-m-e.org/, where a full user manual and guided tutorial are available. PySCeS-CBM and Mariner are also open source, and can be downloaded from http://pysces.sourceforge.net/cbm. FAME and PySCeS/Mariner are covered by their own respective BSD-style licenses, which can be found on the respective web pages and, in short, entail that they are open-source and free to use for both academic and non-academic users.

## Authors' contributions

JB created FAME. BGO created Mariner and PySCeS. JB, BGO and BT wrote the paper. All authors read and approved the final manuscript.
